# Development of a robust real-time synchronized data transmission technique from a Magnetic Observatory to an INTERMAGNET GIN

**DOI:** 10.1038/s41598-022-13820-y

**Published:** 2022-06-18

**Authors:** Sai Vijay Kumar Potharaju, Phani Chandrasekhar Nelapatla

**Affiliations:** CSIR-National Geophysical Research Institute, Hyderabad, India

**Keywords:** Computer science, Information technology, Scientific data, Software

## Abstract

This paper presents the step-by-step development and implementation of real-time data transmission from a distant Observatory. Using Python programming language, we have developed an algorithm for automated data transmission of high resolution real-time magnetic data from Choutuppal (CPL) and Hyderabad (HYB), India to INTERMAGNET Edinburgh GIN, with minimal internet services. We have designed a system to transfer the data in a secured and encrypted pattern with SSH keys and save the same dataset in the local server at CSIR-NGRI. Both the Observatories data are transmitted to GIN in real-time with a time frame of less than 300 s. After successfully transmitting real-time 1 min geomagnetic data from CPL and HYB Observatories to Edinburgh GIN, CPL's 1 s real-time data is also initiated and is one of the first Indian observatories to transmit real-time 1 s data to GIN. The details of algorithm development, function libraries and the challenges faced for 1 s and 1 min data transmission at both the Observatories are discussed.

## Introduction

Geomagnetic Observatories are intended to record long-term observations that provide complete information about the changes in the spatial and temporal characteristics of Earth's magnetic field. As human society becomes progressively technology dependent, there is growing demand from the global scientific community for real-time data from Observatories with a latency period of few minutes, which is a crucial factor in analysing various space weather events, along with satellite data. Employing recent developments in the web-based services for accessing the internet with an end to end secured protocol, many Observatories around the world are making the change to real-time data, while many others are yet to do so. INTERMAGNET stands for International Real-time Magnetic Observatory Network. Its objective, is to establish a “global network of cooperating digital magnetic observatories, adopting modern standard specifications for measuring and recording equipment, in order to facilitate data exchange and the production of geomagnetic data products in close to real-time” was achieved and further improvements continue to be the goal. To date, more than 140 observatories worldwide are producing and transmitting 1 min scalar and vector magnetic data in real-time to INTERMAGNET, which is a world-wide consortium of institutes operating ground-based magnetometers recording the absolute level of the Earth's time-varying magnetic field, to an agreed set of standards.

Many of the INTERMAGNET Observatories (IMOs) are also producing 1 s magnetic field data^1^, with the prominent ones being the 14 Observatories of USGS (United States Geological Survey), 03 Polish Observatories by Institute of Geophysics Polish Academy of Sciences, 04 Observatories maintained by the JMA (Japan Meteorological Agency), 06 Observatories by EOST (École et Observatoire des Sciences de la Terre), 14 Observatories by the GSC (Geological Society of Canada), 16 Observatories operated by IPGP (Institut de Physique du Globe de Paris), 08 Observatories of BGS (British Geological Survey), and 19 Observatories operated by GFZ (German Research Centre for Geosciences), https://www.intermagnet.org/.

Magnetic Observatories have been operational in India for more than 180 years^2^. The prominent ones are Madras Observatory, Shimla Observatory, Trivandrum Observatory (which are not in operation now), Alibag, Jaipur and Hyderabad Magnetic Observatories (in operation till date). The HYB Observatory of CSIR (Council of Scientific & Industrial Research)-NGRI (National Geophysical Research Institute) was commissioned in 1964, and in collaboration with GFZ Potsdam, the 1 min digital three-component fluxgate variation and Overhauser magnetometer data of HYB Observatory was available in INTERMAGNET from the year 2009. The real-time data from HYB Observatory was transmitted to Niemegk Observatory operated by GFZ (Geo Forschungs Zentrum, Germany) by using Linux based shell/bash scripts, and from Niemegk data server, the data was finally transmitted to INTERMAGNET. Another Observatory was established in 2012 at Choutuppal campus, 65 km away from Hyderabad, to study the spatial and temporal variations precisely at a sampling interval of 1 s and achieved INTERMAGNET status during 2019.

The Geomagnetic Information Nodes (GINs) are the collection points for real-time data and are connected to the INTERMAGNET Observatories by satellite, computer and telephone networks. GINs are operating in 5 different countries (i.e., UK, USA, Japan, Canada and France) and use 4 satellites (GOES-E, GOES-W, METEOSAT and GSM) to receive the real-time data from INTERMAGNET Observatories across the globe (https://www.intermagnet.org/gins-eng.php).

There are many Observatories world-wide transfers 1 min/1 s data in real-time to various GINs by using different technologies (Satellite, Telephone ISDN link, FTP, VPN router Servers, in-house build NDL HSS, MQTT and other third-party software’s/tools). To name few:

1. Torta et al.^3^ demonstrated the feasibility of data transmission over 12,700 km link between the Spanish Antarctic Geomagnetic Observatory and Ebre Observatory by using SANDICOM (Sounding System for Antarctic Digital Communications) Ionospheric radio link. Apart from this a Microcom model GTX-1.0 satellite transmitter was also installed at the Observatory and uses the GOES_E link for near real-time transmission and data access from the Ottawa GIN.
2. Data from 13 observatories operated by Natural Resources Canada (NRCan) are transmitting data to Ottawa GIN and other INTERMAGNET data nodes within 12 min of acquisition via GOES satellite^4^.
3. Clarke et al.^5^ developed a proprietary data acquisition logger under the QNX operating system. These data loggers are connected via internet and telephone networks as well as satellite for reliable communication of data from 5 UK Observatories to Edinburgh GIN with few minutes of latency.
4. 14 Observatories maintained by Bureau Central de Magnetisme Terrestre (BCMT) transmits the magnetic data to GIN in near real-time through satellite services. In joint collaboration, IPGP and EOST operate three magnetic observatories and the real-time data from these are available in the INTERMAGNET database within 15 min of acquisition. Reporting delays are due to the practical limitations in transmitting data from remote locations^6^.
5. 5 Observatories operated by BGS transmits the real-time data period of 1 h to GIN via internet and telephone networks as well as satellite communication^7^.
6. Gvishiani et al.^8^ discussed the automated data transfer for Russian National Observatory Network of 9 observatories to GIN by using HSS (holistic hardware/software system) via e-mail or FTP-protocol with a delay time period of 10 min to 2 days depending upon telecommunication capabilities. This data transmission is achieved by setting up a Database server, FTP Server, Webserver, Mail server for the data transmission to GIN.
7. Reda and Neska^9^ transmitted real-time data from 3 Polish geomagnetic observatories with a latency period of 5 min to GIN by via FTP protocol by configuring two VPN router Servers, in-house build NDL (Network Data Logger), Linux server and a backup server.
8. India has a network of 12 Observatories operating by the Indian Institute of Geomagnetism and producing data for the last several decades and for the last few years, the real-time data is being transmitted from 2 Observatories (ABG & JAI) with a latency period of 24 h.

After the cessation of the GFZ-NGRI cooperation for HYB and CPL Observatories, the challenges faced in transmitting the real-time data from these Observatories to Edinburgh GIN became challenging for us. In order to achieve the live data transmission, we have gone through the different techniques and technologies used by the above Observatories, which were expensive, both in terms of maintenance and require huge infrastructure. Upon checking with those Observatories, the data transmission setup was expensive and unaffordable for us to invest. So, we have deployed a limited infrastructure and developed scripts/codes using open-source programming languages/tools to transfer the live data to Edinburg GIN with less than 300 s. The step-by-step approach and the progress made in achieving the task are discussed in the following sections.

## Data acquisition and recording systems

We have deployed Magrec-4B Data Acquisition systems (DAS) at both CPL & HYB Observatories for recording the vector data from DTU-FGE magnetometer and scalar data from Overhauser GSM90-F1 magnetometer. The Magrec-4B data acquisition system, manufactured by Mingeo Ltd (Hungary), is a dual-core Intel® AtomTM-Processor-based small-size fan-less data acquisition computer equipped with a PalmAcq GPS timing module (http://www.mingeo.com/prod-magrec4b.html) and is deployed by many Observatories around the world. Magrec-4B plays a vital role in storing the GSM90-F1 data, which does not have any internal memory, and in tracking the time-stamping differences between the fluxgate magnetometers if any. Magrec-4B also provides information about the short- and long-term real-time plots of acquired and filtered data, logs of different events and errors of the recording systems, and information about the unexpected shutdown of the systems if any.

However, Magrec-4B has very low disk space; bandwidth and chances of disk failures are high, necessitating the arrangement of more robust data storage system in each Observatory. Using bash/shell scripts, we have configured the Magrec-4B DAS to transfer the data to Linux a high-end workstation with large storage and robust Linux OS for further examination and processing of the data. We have also written shell scripts in both the Linux machines located at both the Observatories to transfer the data to the Server located at HYB Observatory.

## Stage wise implementation

The initial trials of transmission of 1 min magnetic data from HYB and CPL Observatories to INTERMAGNET Edinburgh GIN were started in mid-2018. The details of stage wise implementation of data transmission from both the Observatories are discussed in subsequent sections.

### Data uploading by Email

Firstly, the option adopted was to Email (e_gin@mail.nmh.ac.uk) the data on an hourly basis. For this option, we need not send a whole day file but can send the data that has been recorded since the last sent data to the GIN. If the data was to be divided into hourly blocks starting at the start of the hour (i.e., containing minutes 00–59 for only 1 h), it is easy to use the IMF (INTERMAGNET Minute Mean Format), which requires a minimum of 1-h of data, since the data is batched in hourly blocks. Subsequently several efforts were implemented to optimize the data delivery route. For sending one full day record by Email from both the Observatories, we have two ways:

- the email message (subject line) should have an IAGA-2002 data file format attached with a valid filename.
- the IAGA-2002 data file should be in the body of the Email, and the Email must be in plain text, with no formatting or additional text. If one uses this method, the Email's subject must be set to the IAGA-2002 filename, with no additional characters.

With modern Email systems, it is challenging to achieve step-b (data in the body of the Email message), so it may be easier to send the data as an attachment (step-a). In real-time, sending Emails and monitoring the same every day was a difficult task.

### Data uploading via the upload form

**Figure 1:**
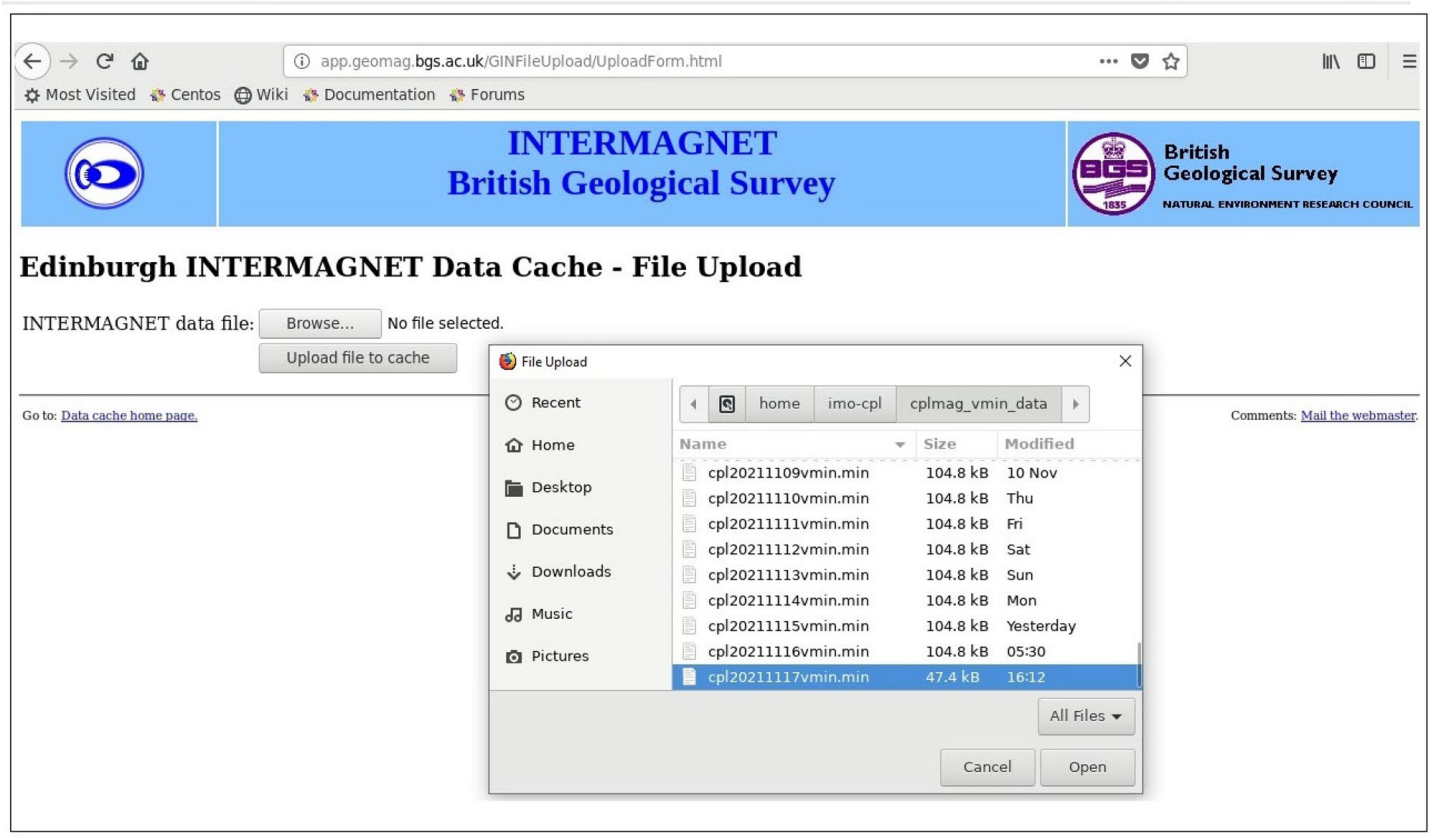
Email upload form for manual uploading of each day’s data file via the BGS website.

The alternative method to the Email was to upload the data file via the BGS upload form website (http://app.geomag.bgs.ac.uk/GINFileUpload/UploadForm.html) (Fig. 1). For the IAGA-2002 data format, there is no issue about where the data starts and ends. The data can be sent at any arbitrary length in a file since IAGA-2002 includes a timestamp for every data record. It is easier to send parts of a day's data using IAGA-2002 format, but it is also possible to use the older IMF.

The software at the GIN will overwrite any non-missing data values in the file that it receives. If we send data in parts of a day, either through the web or Email, the Edinburgh GIN will reassemble the data into a day file and forward the day file to the INTERMAGNET website for distribution to the users. There are several instances where the data upload to GIN got interrupted due to limited human resources, since the job requires a person to do it exclusively every day irrespective of other committed assignments (magnetic surveys, field works, attending national and international symposiums, etc.).

### Data uploading by an automated process

Using CURL, JAVA and UNIX shell scripts, BGS and INTERMAGNET developed automated process of uploading data files to the data cache, searching the cache, and downloading data from the cache. The web interface provided by BGS allows users to deposit data within the GIN in an automated way using the 'curl' or 'wgets' commands. For more information, visit http://app.geomag.bgs.ac.uk/GINFileUpload/HelpAutomate.html. One needs a username and password to access this site, for example, ABC/XXX.

Initially, we tried to use the 'curl' or 'wgets' commands for depositing the data to GIN, but we have encountered errors such as HTTP Protocol and Authorization error; the username assigned was being treated as a host rather than a user. The ‘curl’ command was getting mangled by the script; several other errors could not be fixed because of platform dependency at our end. Moreover, troubleshooting the issues, data transfer, time zone differences, and limited human resources at our end would result in delay of transmission of the live data to the GIN. To resolve the issue at the earliest, we also needed to rely on a BGS expert, which could be a time-consuming process, as the experts might be occupied with their commitments and with other Observatories data transmission processes.

To overcome all the above issues of sending the data by Email upload form/automated upload to INTERMAGNET BGS website with the log-in credentials, we have developed a Python code to upload the data in automated technique, which allows us to monitor if any issues arise and enables us to understand the errors by checking the logs at our end during data transmission.

We found Python to be more suitable compared to other commercial software like Matlab, MQTT, JAVA and other Cloud Technologies, which would need dedicated storage in the host machine and also involves commercial aspects like purchasing, maintenance, and upgradation of the license. Whereas Python is an open-source software, and it has many advantages like writing the code in few lines, easy to update/upgrade, and no need to compile the code. Python also has the capability to scale quickly to solve any complex problems making it a programming favourite. It is easier to develop and debug at any given time and in any scenario and can be used for future developments or upgrades, not just for data transmission but for many other significant purposes.

The details of the developed automated robust data transmission technique will be discussed in the subsequent sections.

## Setting up of indigenous data transfer protocol & transfer service scenarios

Since the internet availability at CPL is very limited due to its location far-off from a city, we have approached a reliable permanent fiber optic setup from BSNL (Bharath Sanchar Nigam Limited) maintained by Govt. of India. But it was too expensive to set up and maintain, so we have used the facilities of a local service provider, with the maximum bandwidth of 20 Mbps to initiate the data transfer technique.

### Initial configuration

The online data transfer from CPL to HYB Observatory was started using cross-platform data transmission as the ISP (Internet Service Provider) resources were unavailable. As the service provider could not resolve a few issues at the TCP/IP network level about data transmission from a Linux machine to another distant Linux machine, we had to go ahead with a cross-platform data transmission process, since the final data had to be processed on Windows based Matlab codes.

**Figure 2:**
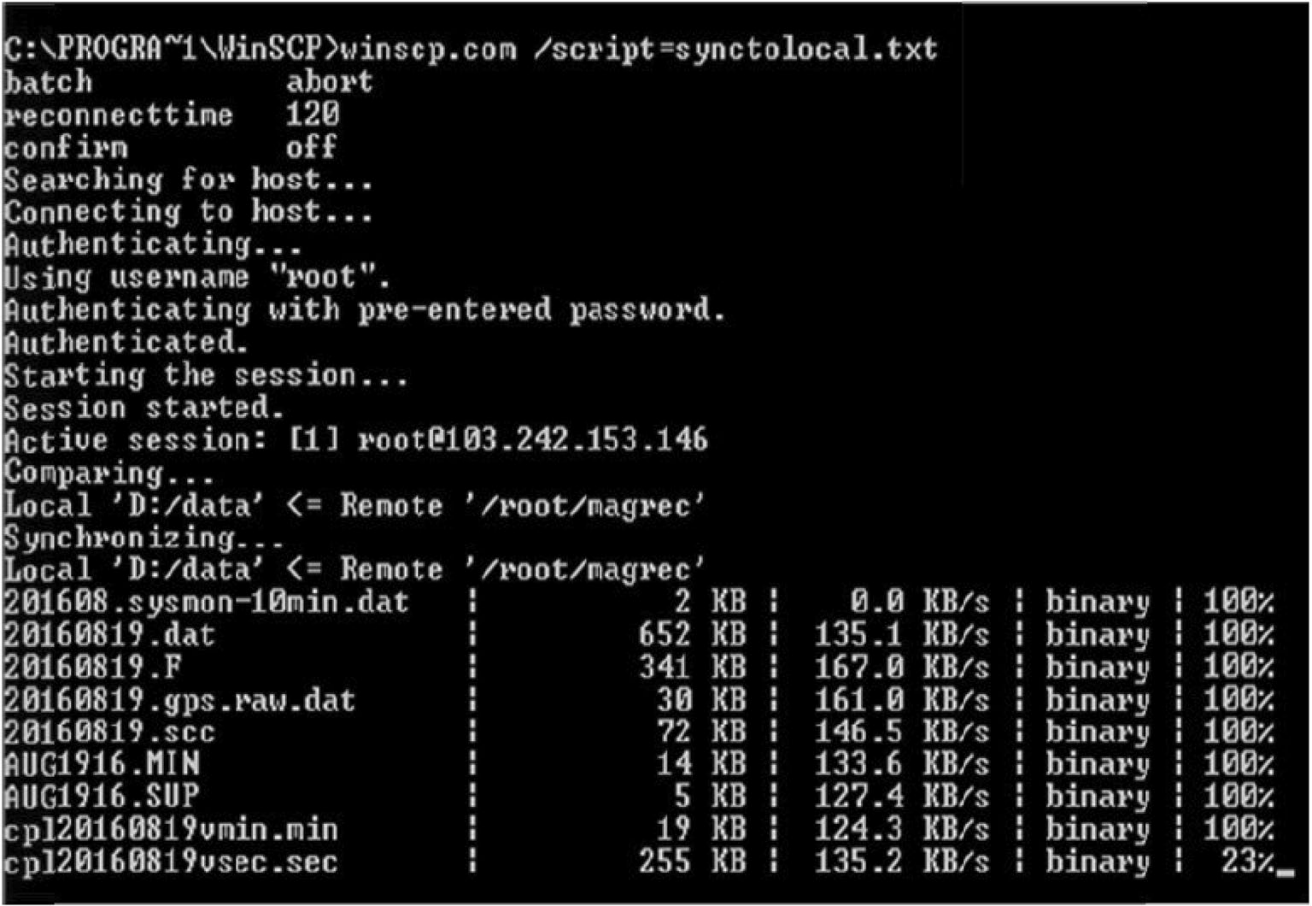
Cross platform data transfer system from Linux PC (deployed at CPL) to Windows PC (deployed at HYB) & percentage of successful data transmission between the systems.

Initially, we have set up some shell scripts, cron jobs and rsync protocol to transfer the data from the Magrec-4B data logger to an intermediate Linux machine (Centos) deployed at CPL. The data was transferred from Magrec-4B to the Linux machine (backup storage) at the CPL control room with a latency of 5 min and then it was transferred to a Windows machine (client) at HYB Observatory using codes, scripts developed by us and, third-party tools (Fig. 2). Since the bandwidth was low, we have decided to transfer the data from the Linux machine to Windows pc at HYB-NGRI with a time-lapse of 1 min.

We have installed a Batch file with the "Abort" option and confirm with the "Off" option to ascertain the health of the connection at the client end (Windows pc), reiterated for a default time limit of 120 s. The session commences by checking the Host ID username and the authenticated pre-entered password with RSA (Rivest, Shamir, and Adelman) Key through SFTP (Secured File Transfer Protocol). The terms ‘Comparing’ and ‘Synchronizing’ in the figure show the details of the data transmission from host to client machine at conventional intervals with a time interval of 120 s.

From Magrecc-4B, we have culled 9 data parameters as shown in Fig. 2, to transmit real-time data to the Client machine. The details of the file size, each data parameter, and the haste at which the data is being transmitted from the host to the client machine are withal. The percentages in column 5 of Fig. 2 show the client machine's data transmission and updating process. 100% data transfer is achieved only when the data is copied with the latest records of 120 s, and additionally, the client machine rechecks the data by synchronizing the earlier records of the current day. The example of the perpetual data transmission process with the latest records and the updating process is also shown in row 9 of Fig. 2. Once the data is synchronized with the latest records (for example row 9 filename in Fig. 2), the 23% of file transmission will become 100% upon completion of this task, further synchronizing with the earlier saved data. The file size of the above said nine parameters keeps incrementing for every 120 s of the data being updated at the host machine. The whole process is reiterated for each cycle of 120 s till the day is completed.

As a large amount of data from both the Observatories is to be transferred and needs a dedicated storage to save the data on a daily basis, we have setup a server at HYB Observatory. And also, at CPL, the internet network services were upgraded recently along with the increased bandwidth of 50 Mbps (which is the maximum available bandwidth to date), which allowed us to configure the automated robust data transmission technique to GIN and details of these are discussed below.

### Final configuration

Since our main aim was to achieve automated 1 min data transmission from HYB and CPL Observatories to GIN, we had to make additional R&D efforts to develop a robust setup concerning both hardware (i.e., high-end workstation, firewall router setup) and software. Thus the Python code, shell scripts, cron jobs and rsync protocol were developed to take care of data transmission without data loss. Even when there is a disconnection in the internet services, once the internet services are restored, the Python code will recheck the data from the last successful transmitted file.

The data transfer from CPL and HYB to the Central Server located at HYB Observatory, does follow RSH & SSH key algorithm which is by itself a much secured algorithm. We have designed a system to transfer the data in a secured and encrypted pattern with SSH keys and save the same dataset in the local server at CSIR-NGRI. We have used the RSA-SSH algorithm (Rivest–Shamir–Adleman), which is a public-key cryptosystem that is widely used for a secure data transmission. The key generated by the ssh-keygen in the source machine (MAGREC-DAS) will create two files namely “id_rsa & id_rsa.pub in the .ssh directory, which is shared/copied to the destination machine (Centos). So there is a perfect handshake in between the source and destination machine for data transfer. This setup remains the same unless the network remains the same, so for this reason we have assigned a static IP. Along with the ssh-keys, a code has been written to transfer the data using ‘rsync tool’ and the same has been inculcated in the ‘crontab’ to keep reiterate the same with a time frame of 10 s. Now the same technique was also used at HYB Observatory from Centos machine to the Server for a secured and successful data transmission.

**Figure 3:**
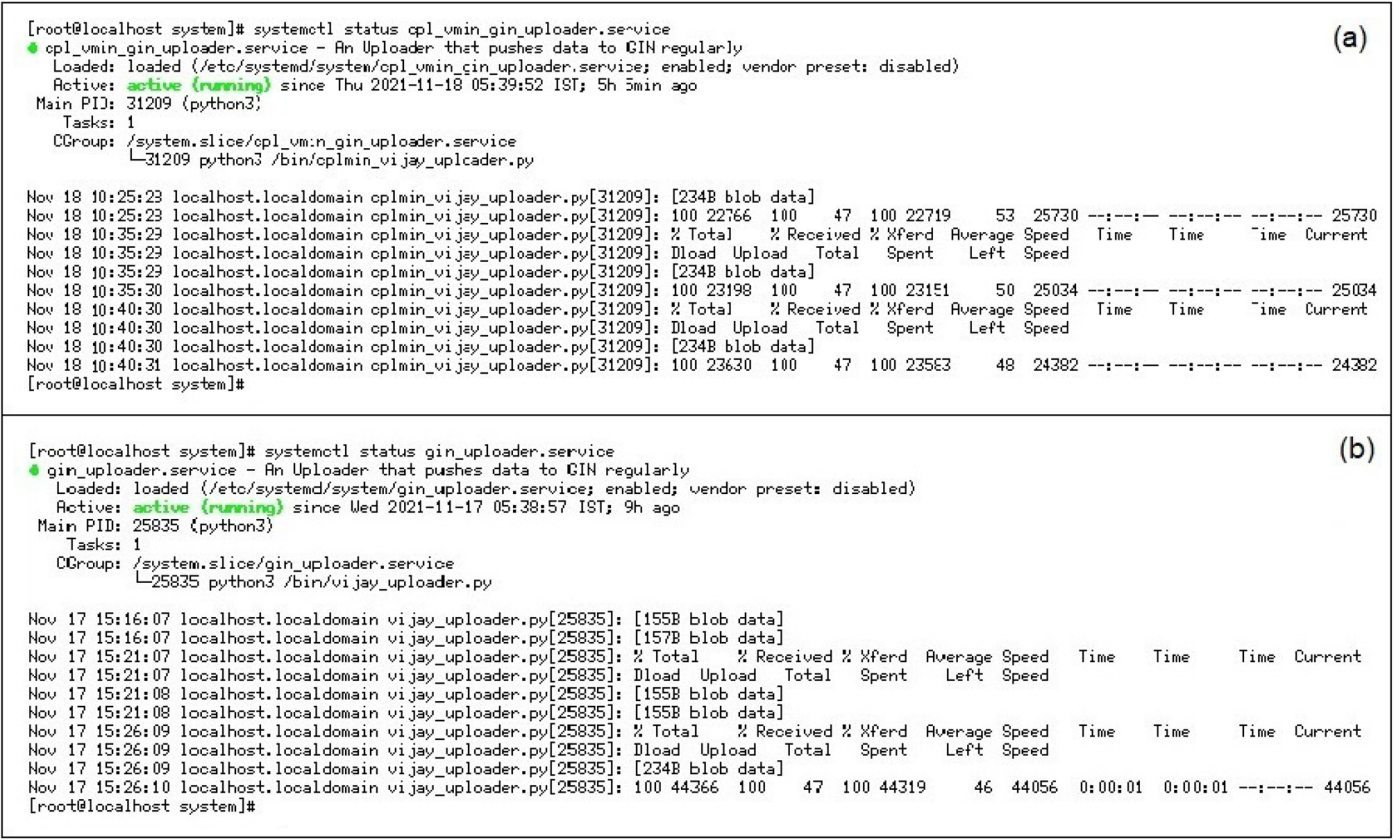
Automated 1 min data transmission from (**a**) CPL and (**b**) HYB Observatories to Edinburg GIN using the Python code.

After the successful R & D efforts of data transmission from both the Observatories to a dedicated high-end Linux Server, with a 24 TB RAID-5 configuration at HYB Observatory, we have created individual user accounts in the server, i.e., IMO-CPL, IMO-HYB, to store the received data from the respective Observatories. The developed Python code will transfer several data types from DAS and store them in respective user accounts daily (Fig. 3). The developed scripts from each Linux PC will filter the data according to the directory's requirement (i.e., GIN). The sorted data from the individual directory will be transmitted with a latency period of 300 s to INTERMAGNET GIN.

**Figure 4:**
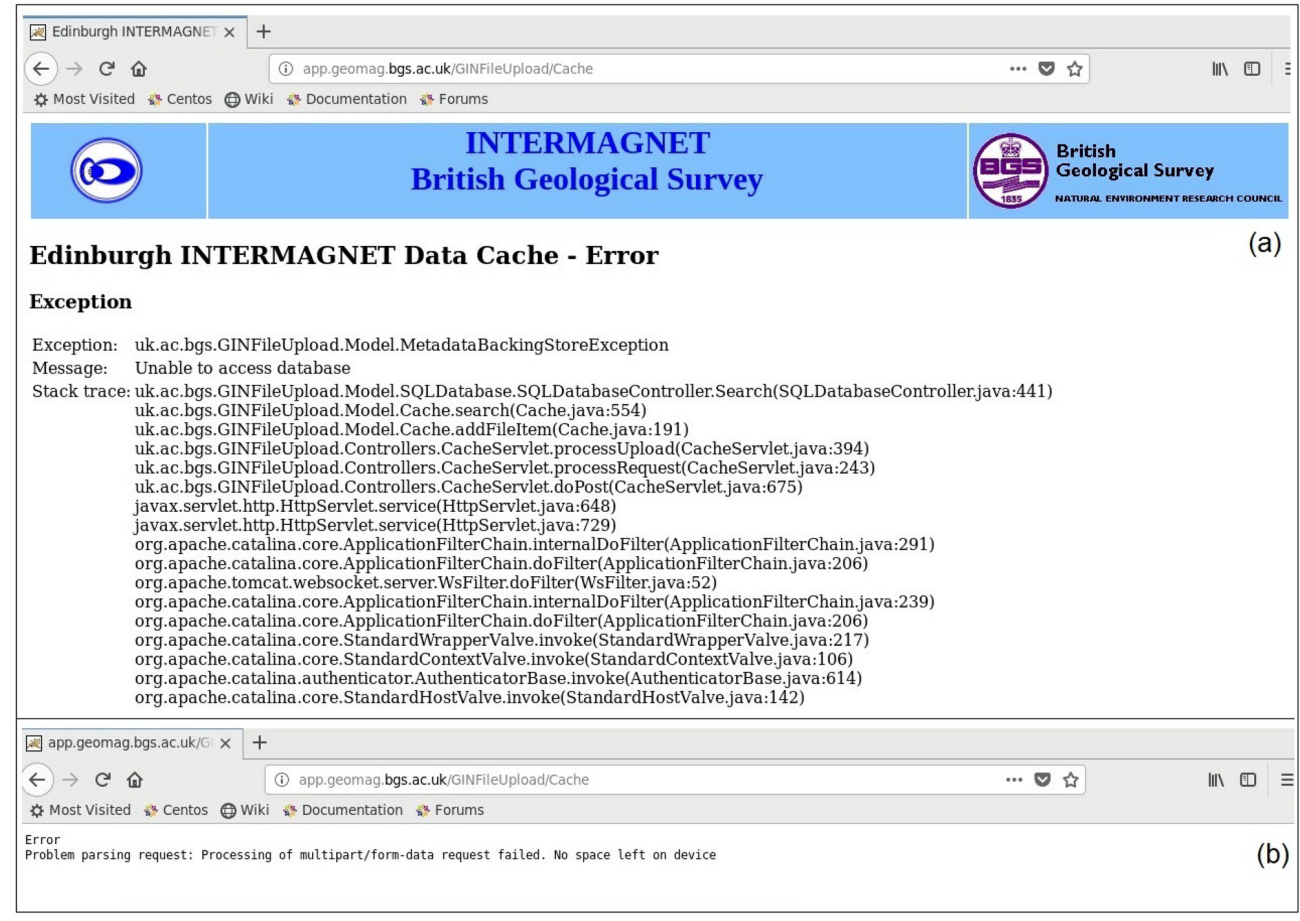
(**a**) Details of data cache memory for both the Observatories at INTERMAGNET BGS website (**b**) error message showing “no space left” due to huge volume of duplicate data on BGS server.

After successful data transmission from both the Observatories to GIN, we came across a few minor issues, and how we have resolved them is discussed in detail:

**Issue-1** Initially the Python code was executed using ‘rsync synchronization protocol’ with a minimum latency period of 60 s to transfer the real-time data from both the Observatories. As reported by GIN experts, with this latency period the same data was being sent repeatedly to the receiving web service (http://app.geomag.bgs.ac.uk/GINFileUpload/UploadForm.html), Fig. 4a, due to which the storage/cache memory at GIN was receiving huge volumes of data from both the Observatories. This was causing problems for their entire web service, with log files filling up very quickly and, the cache of the data in the web service was difficult to use because it occupies large disk space (Fig. 4b).

**Solution** To resolve the above issue, we have created background daemons instead of 'rsync synchronization protocol', so that the rechecking of data for every 60 s was replaced with 300 s. The daemons at the backend will execute the Python code for every 300 s for smooth data transmission of real-time data without any duplication (as shown in Fig. 3).

**Issue-2** After successful transmission of data from both the Observatories, on a few occasions, the data plotting services at the INTERMAGENT were not reflected even though our hardware and software were intact. We have cross-checked the logs from our end and found the data was successfully uploaded to GIN. Even though the data records are successful, why was the data not plotted on the INTERMAGNET website was unknown.

**Figure 5:**
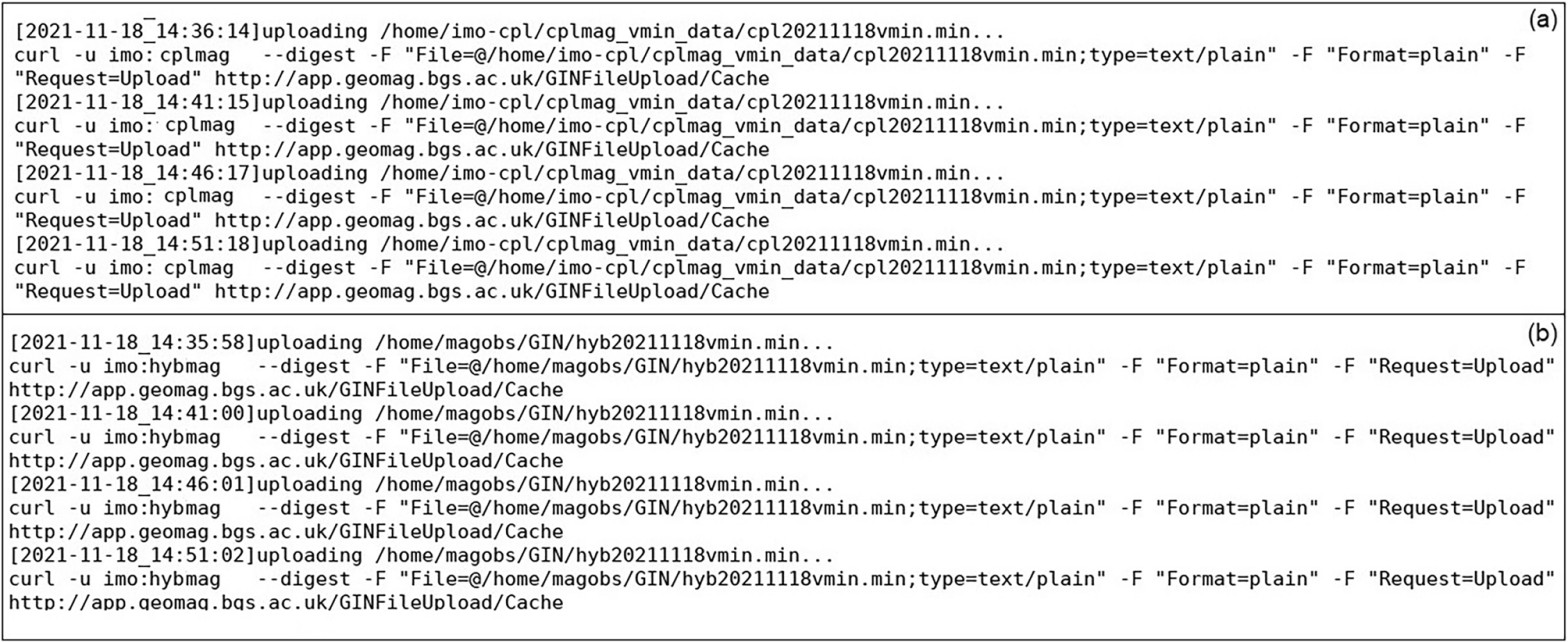
Cross-checking of (**a**) CPL and (**b**) HYB data logs from HYB Observatory sever to GIN server.

**Solution** The above issue was resolved after the BGS experts have suggested a link (http://app.geomag.bgs.ac.uk/GINFileUpload/UploadForm.html) to upload a single day file to check if it was successful or not? As suggested by BGS, if the upload of data was not successful and with some errors (Fig. 4), there is an issue at the INTERMAGNET server. This verification made us ascertain that the code which we are executing is functioning correctly (Fig. 5).

## Discussion

In general, most of the Observatories are located far away from the cities to record and produce high quality noise free magnetic data, whereas servers are located at certain data centers having good network with high speed internet bandwidth, storage, strong security firewall devices and uninterrupted power supply, therefore, reliable transmitting data from the rural area with constraint bandwidth and unpredictable weather with limited budget to data centers is one of the critical missions to accomplish.

Given that the near real-time data transfer today is feasible, it requires integration of hardware, software, and user training to efficiently monitor the data transmission system.

Clarke et al.^5^ and Gvishiani et al.^8^ discussed the conventional file transfer protocols (such as FTP, mail, Rsync) for transferring the real-time data from Observatories to GIN. All these transfer protocols (ex. Rsync, FTP, SFTP and mail) are good at domains but none of them actually implement incremental uploading for specific files, elastically change configuration, logging system, retry mechanisms and easy migration of the program to another host on demand.

Apart from limitations, deploying and setting up a Database server, FTP Server, Webserver, mail server that fulfils the above characteristics for the data transmission to the Observatories as well as to GIN. But it needs rigorous setup and also requires a lot of financial investment followed by maintenance and disaster recovery of all the servers is a challenging task. Reda and Neska^9^ configured two VPN router Servers, in-house build NDL (Network Data Logger), Linux server and a backup server for transferring real-time data to GIN. Setting up a VPN router service requires a huge setup and is expensive. Many others discussed the feasibility of real-time data transmission from magnetic observatories to GIN via satellite communication^3–7^. Data transmission by satellite communication is very fast but it is expensive.

There are numerous programming languages (ex. C, C +  + , JAVA, and etc.) and commercial tools (Matlab, AMQP, STOMP, MQTT and etc.) able to transfer the data in real-time to GIN. Even with the available commercial applications, high-end hardware and internet facility, one need to have a considerable amount of acquaintance with any of these languages and corresponding operation systems because whenever there is a failure in data transmission one needs to have hands-on experience to resolve the issue and resume the data transmission in no time.

Keeping in view of all the above constraints (file transfer protocols, satellite communication, commercial software’s and other resources), we have come up with a low-cost robust real-time data transmission technique by developing a Python code and a Linux daemon with very limited resources (hardware and internet services). The advantages of the developed Python code are discussed below:

There are 3 levels of services: infrastructure, platform and software as a service. As described above with a very minimal infrastructure we have set up the entire data transfer modules, right from the security features (Firewall), ISP, Servers, Workstations (backup storage) and etc.

We have chosen Linux as our underlying platform for storage, backup server, programming and data transmission. As an Open-Source operating system we can deploy, update and install all the necessary software’s without any investment. The maintenance part of the Operating System is also very less.

The core logic of the data transmission program is written in Python3, one of the most popular programming language for more than a decade, is because it is open-sourced, available out-of-box in Linux and as a scripting language one can write and test the code with spending time on compiling, linking and deploying; the performance is very close to native code, with respect to these characteristics, maintaining and adding new features are very handy.

The code which we have developed has the below robust features which have been the key features to transfer the near real time data to GIN.

1. Reliability: It periodically sends the data by checking the TCP ACK from the Observatory to GIN and vice versa.
2. Smart selection: It differentiates the sent data by checking any duplicate data at the source machine. Each day's data file will be sent with a latency period of 300 s. It will never introduce duplicates, it can be referred as “fire and forget”. By all means it's fastest and consumes the least band-width to transfer the data to GIN.
3. WatchDog logging: It logs every circumstance, from success to failure, from data inexistence (no new data) to data accumulation.
4. Elastic and secure configuration: whenever we want to change the uploaded directory, login password or the period of uploading duration, simply by changing the config without restarting the program.
5. Daemonization: We have established a system daemon that invokes the code whenever the system boots up and auto-restarts & whenever the service goes off.
6. Rolling deployment: Whenever we enhance the code, we just issue a "git pull" and restart the daemon, which takes ~ 3 s to finish.
7. The greatest thing is the design part of our code which takes care of all the preliminary and the most important steps in a real time data transmission.

Apart from all the above, the data transmission is stopped when there is a network disconnectivity and the connection is re-established the data is automatically resent from where it has stopped transmitting. We have also established the connection between the systems at each Observatory so that even if there is a data connection lost at CPL to HYB Observatory due to interruption in internet services, the data will be transmitted from MAGREC to Linux machine which are connected locally through fibre optical cable. So, we are not losing the data at any point in time, and once the internet connection is re-established, the data transmission will restore from where it has stopped. To avoid packet loss over long distances data transmission, we have increased the latency period to 300 s and made sure the same data is not repeated at the GIN server as duplication and filling up the cache memory.

**Figure 6:**
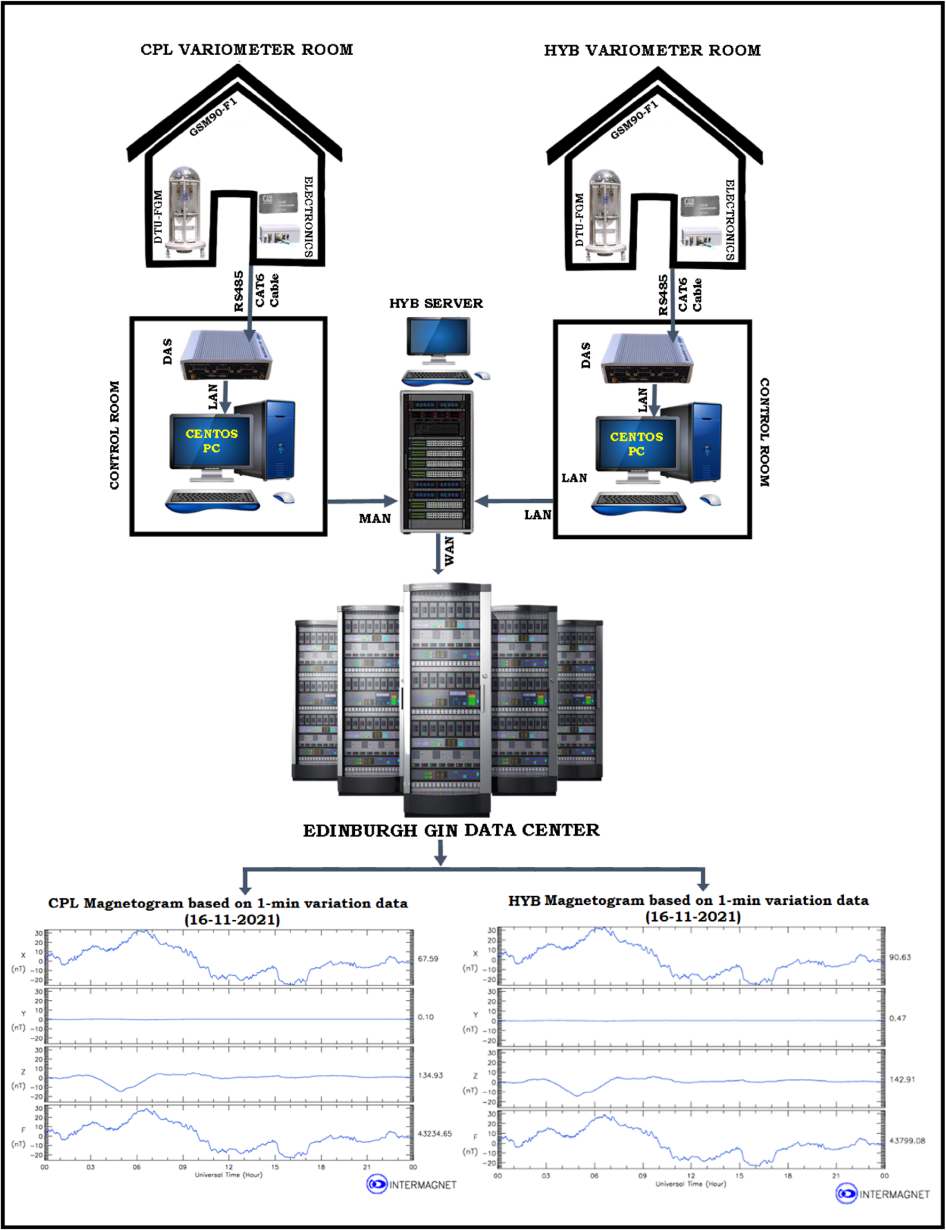
Schematic diagram showing the recording setup at CSIR-NGRI Observatories (CPL & HYB), data transmission to Edinburgh GIN, and real-time magnetograms of the Observatories on the INTERMAGNET website.

As discussed in Sect. 2, the details of scalar and vector recording systems, DAS, and data transmission from CPL to HYB Observatory and from there to INTERMAGNET GIN are shown in Fig. 6. The real-time data from both the Observatories transmits the data to GIN with a latency period of 300 s. The 1 min real-time magnetograms of H, D, Z, and F components from these Observatories are also shown in the Fig. 6.

## Transmission of 1 s data from CPL Observatory

**Figure 7:**
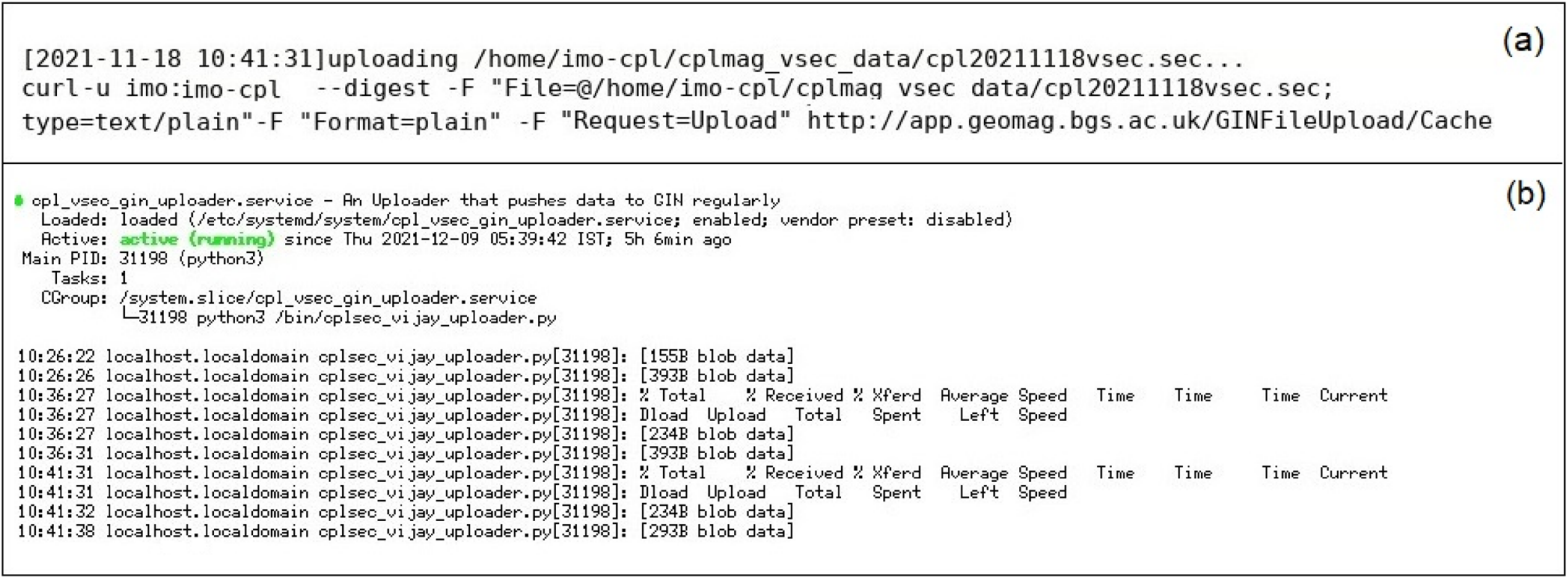
(**a**) Details of 1 s data transfer logs of CPL Observatory to GIN (**b**) details of successful running (active) of Daemons running in the background and 1 s data transfer from CPL to HYB and to GIN.

**Figure 8:**
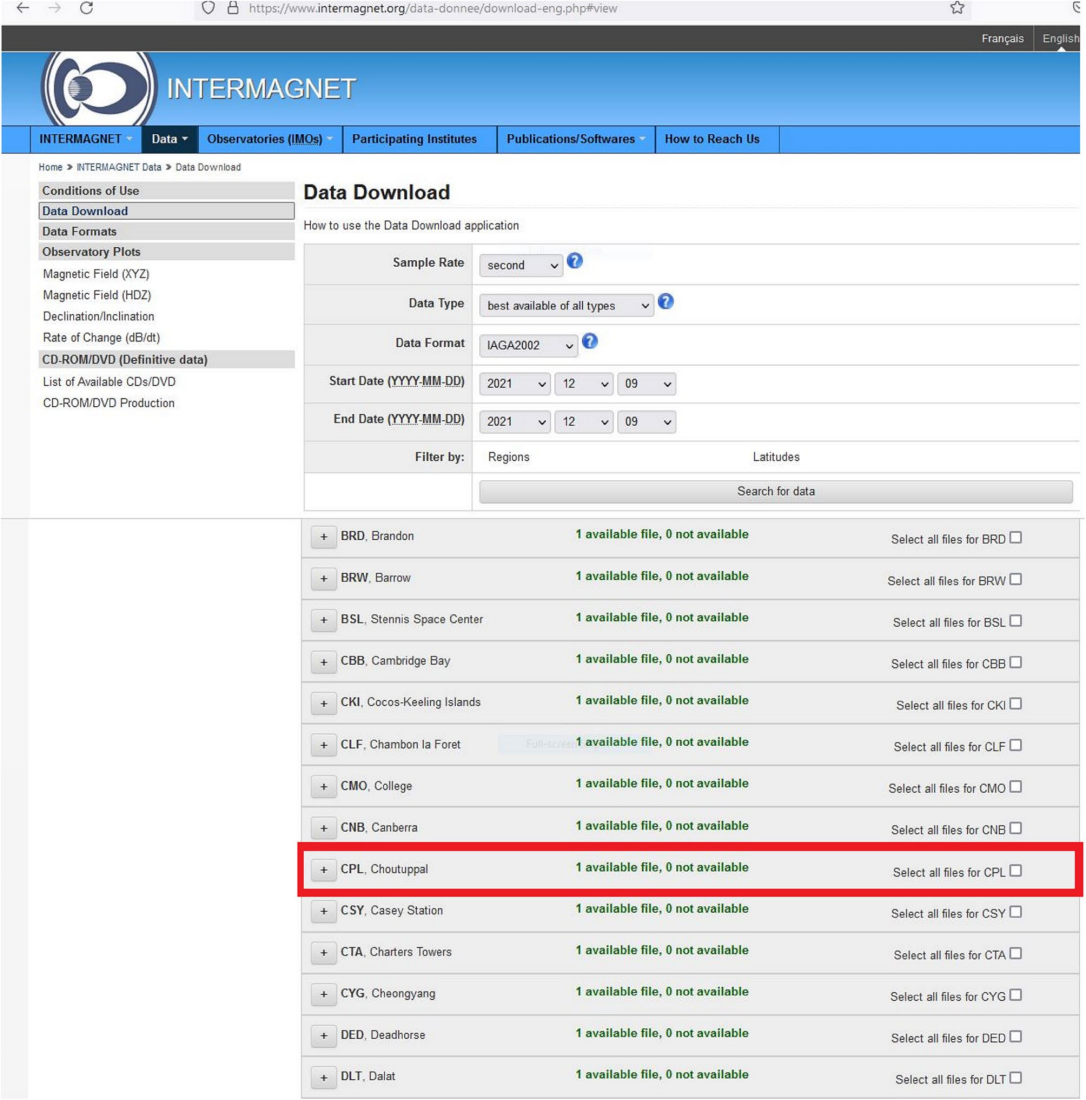
Availability of 1 s data of CPL Magnetic Observatory on INTERMAGNET website.

After achieving the successful 1 min data transmission to GIN from HYB and CPL Observatories, we have started working on the data transmission of 1 s to GIN by taking utmost care with the issues we have encountered while transmitting the 1 min data (ex: data duplication, latency time frame, python code, scripts and tracking of logs). Figure 7a shows the logs of 1 s data transmission GIN Fig. 7b daemons running at the background and successful 1 s CPL data transmission to GIN. Figure 8 shows the availability of CPL 1 s data on the INTERMAGNET website.

The successful data transmission from CPL and HYB Observatories to GIN has been achieved with less expenditure, unlike investing a lot in satellite-driven devices to transmit the data. There are many other alternatives like cloud sharing, data transmission through satellite, Etc. which are all cost-effective. Cloud usage may be limited because of regulation, the enormous size of the files, and the slow sharing speed. Cloud can get expensive, which could be a limiting factor as well.

## Conclusions

This paper presents the successful implementation of 1 min real-time data transmission from both the CPL and HYB Observatories based on the robust python programming code with persistent connection, and it uses this scheme in an application of a network management system with a minimal and cost-effective approach. This technique implements the push mode to the web server of GIN and has good applicability and versatility to transfer real-time information. The firewall does not restrict the transmission, and the user does not need to install additional plug-ins. These features ensure the security of information transmission.

The challenges faced for 1 min and 1 s have given us a way to use the resources significantly, which any other Observatory may find useful. This approach helps in the evolution of new services; we could try out new protocols with the upcoming advanced features in the current Linux operating systems and Python programming to reduce the latency period less than 300 s. We believe that this cost-effective approach of high-quality data transfer with minimal resources could provide significant benefits to researchers, users across the globe, and the scientific community.

## Data Availability

The datasets generated and/or analysed during the current study are available in the [CPL/HYB] repository, [https://www.intermagnet.org/data-donnee/download-eng.php#view]. The transmitted 1 min (HYB & CPL) and 1 s (CPL) magnetic field variation data are available in the public domain (https://www.intermagnet.org/data-donnee/download-eng.php#view). These Observatories data can download by selecting the respective Observatory (CPL/HYB) in the search option by providing a valid E-mail id to download the data. Any further material information will be available from the corresponding author on request.
